# On the epigenetics of vascular regulation and disease

**DOI:** 10.1186/1868-7083-4-7

**Published:** 2012-05-23

**Authors:** Christina Schleithoff, Susanne Voelter-Mahlknecht, Indra Navina Dahmke, Ulrich Mahlknecht

**Affiliations:** 1 Saarland University Medical Center, Department of Internal Medicine, Division of Immunotherapy and Gene Therapy, Homburg, Saar, D-66421, Germany; 2 Institute of Occupational and Social Medicine and Health Services Research, University of Tuebingen, Wilhelmstrasse 27, D-72074, Tuebingen, Germany

**Keywords:** Epigenetics, Cardiovascular disease, Vascular regulation, Sirtuins, Histone deacetylase, HDAC

## Abstract

Consolidated knowledge is accumulating as to the role of epigenetic regulatory mechanisms in the physiology of vascular development and vascular tone as well as in the pathogenesis of cardiovascular disease. The modulation of gene expression through modification of the epigenome by structural changes of the chromatin architecture without alterations of the associated genomic DNA sequence is part of the cellular response to environmental changes. Such environmental conditions, which are finally being translated into adaptations of the cardiovascular system, also comprise pathological conditions such as atherosclerosis or myocardial infarction. This review summarizes recent findings on the epigenetics of vascular regulation and disease and presents nutritional and pharmacological approaches as novel epigenetic strategies in the prevention and treatment of cardiovascular disease.

## Introduction

Epigenetic alterations are chromatin-based modifications that affect the expression of genes without altering the DNA sequence itself. Such modifications include the methylation of DNA, the posttranslational modification of histone proteins and RNA-based mechanisms, [1] which may altogether modulate the tertiary structure and thus the accessibility of promoter DNA for transcription factors and numerous regulatory elements that finally affect transcription [2]. Epigenetic processes are essential as to the determination of cell identity and for the propagation of modifications that are meiotically and mitotically heritable [3]. In this context, epigenetic pathways are key elements in the regulation of endothelial gene expression, and thus in the pathogenesis of vascular disease such as atherosclerosis and vascular restenosis. The vascular system is highly flexible with regard to physiological and pathological challenges and carries therefore the potential to regenerate and generate new structures during the whole organismal life span on the basis of two major epigenetic principles: the heritable propagation of the information on changes in gene expression without alterations in the DNA sequence, and the capacity to develop different phenotypes from one single genotype [4].

## Epigenetic regulation - overview

Since Conrad Waddington first coined the term “epigenetics” back in 1942 as the study about “the causal interactions between genes and their products, which bring the phenotype into being”, research progress since then led to an advanced definition [5]. More recently, epigenetics was redefined as the study of “stable alterations in gene expression without alterations in the genetic code itself” [6]. These alterations in gene expression are achieved by changes in the tertiary structure of the DNA strand and thus the accessibility of the DNA for molecules which effect gene expression. Under physiological conditions this machinery allows relatively fast heritable changes in gene expression in response to environmental factors such as nutrition or lifestyle conditions and plays a pivotal role in embryogenesis and genomic imprinting. Accordingly, the addition of activating or repressive epigenetic marks affects gene expression. The permanent silencing of one X-chromosome in women as an example is explained by heavy genomic DNA hypermethylation of CpG islands [7]. Significant alterations of the epigenome may be found in numerous diseases such as asthma or cardiovascular disease and different types of cancer, which highlights the potential of epigenetic modifications on the development of novel epigenetic treatment strategies.

The tertiary DNA structure is composed of chromatin, a complex of DNA, histones and other chromosomal proteins. The nucleosome is the fundamental component of chromatin and consists of a protein octamer that contains two copies of each of the histone proteins H2A, H2B, H3 and H4 with and 146 base pairs of DNA, which are being wound around this protein core [2]. Such a fibre of sequentially arrayed nucleosomes and linker histone proteins is preferentially arranged into a stable 30 nm solenoid tertiary structure, which is itself the basis for stable higher order chromatin compaction [8]. The degree of chromatin condensation is reversibly regulated by epigenetic mechanisms and thus, chromatin may principally be found in two variants: euchromatin and heterochromatin. Euchromatin stands for decondensed chromatin, i.e. transcriptionally active chromatin. Heterochromatin on the other hand, describes highly condensed and therefore transcriptionally inactive chromatin [2] Activating epigenetic marks are therefore predominantly found on euchromatin, while heterochromatin is primarily associated with repressive epigenetic marks [9].

### DNA methylation

DNA methylation is one of the fundamental epigenetic marks that is associated with transcriptional silencing, and plays a key role not only in X-chromosomal inactivation, but also in embryonic development, genomic imprinting, and lineage specification [1,10,11]. In mammals, DNA methylation takes place mostly within CpG dinucleotides, where a methyl group that is derived from S-adenosyl-L-methionine is bound in position 5 within the cytosine ring, thus forming 5-methyl-cytosine [4,11]. This pyrimidine can still constitute a base pair with guanine [1]. In healthy somatic cells, up to 90% of CpG dinucleotides, i.e. 3% to 6% of all cytosines, are methylated, except for promoter CpG islands, which appear to be protected from methylation [7,11].

So far, three active DNA methyltransferases (DNMTs), which catalyse the methylation of mammalian DNA cytosines in position C5, have been described: DNMT1, DNMT3a, and DNMT3b [1] DNMT3L which belongs to the group of DNMT proteins lacks the catalytic activity itself, but is required for the enzymatic activities of DNMT3A and DNMT3B [12] DNMT2 is a highly conserved protein and shows a strong tRNA methyltransferase activity. It is known to methylate aspartic acid transfer RNA without being restricted to this process [13]. Amino acid methylation of arginine and lysine residues of proteins is usually carried out by peptilarginine or lysine methyltransferases, respectively. During embryonic development, DNMT3a and DNMT3b are responsible for *de novo* methylation, and for the creation of specific DNA methylation patterns [11]. While DNMT3a is essential in the context of genomic imprinting during gametogenesis, [14-16]. DNMT3b plays an important role during embryonic development [15]. DNMT1 is required for the maintenance and propagation of DNA methylation patterns across generations of cells, i.e. the methylation pattern is carried along the replication process during mitotic cell division [1]. DNMT1 is however not that efficient as to the maintenance of methylation patterns within CpG dense regions [11] and during massive demethylation events such as after fertilization, [14] which may as a consequence lead to alterations of the original methylation pattern. Three principal mechanisms are involved in the regulation of gene repression via 5-methyl-cytosine: First, 5-methyl-cytosine can sterically affect transcription factors to their *cis*-DNA binding elements, as it is described for several transcription factors [17-20]. During this process, methyl groups of methylated CpG dinucleotides intercalate into the major groove of the DNA helix [1]. Second, methyl-CpG binding proteins such as MeCP2, interfere with the recruitment of transcription factors (i.e. DNA-binding *trans* factors) [2]. Third, these methyl-CpG binding proteins are able to recruit large protein complexes which control the accessibility of DNA through modification of the chromatin structure [11]. On the other hand, a transcriptional activator, human CpG binding protein (hCGBP), has been reported that specifically recognizes and binds *un*methylated GpG dinucleotides [21]. Even though, both, active (replication-independent), as well as passive (replication-dependent) mechanisms have been described, the process as of how DNA methylation marks are exactly removed, is still unexplained [11]. Therapeutically, nucleoside analogues require proliferating cells in order to inhibit DNA methylation, because the nucleoside analogues exert their activity through an irreversible covalent bond with DNMTs after their incorporation into DNA [7].

### Histone proteins

The histone proteins within the nucleosomal core consist of a globular domain and an N-terminal tail, which may be subjected to more than 60 variations of posttranslational modification [2] .Lysine for example, can be modified by acetylation, methylation, ubiquitylation or sumoylation, while arginine can be modified solely by methylation; and finally serine and threonine by phosphorylation [10,22]. In contrast to DNA methylation, most histone posttranslational modifications are highly dynamic processes [1]. Even though some of these covalent modifications take place within the histone globular domains, the best studied modifications – and more specifically the effects of lysine acetylation and methylation on chromatin condensation and thus on the regulation of the activity of gene promoters, are within the histone N-terminal tails [10,22,23].

### Histone acetylation

Histone acetylation goes along with transcriptional activation and is mediated by histone acetyltransferases (HATs), [1,2,24] while it is antagonized by the opposing histone deacetylase (HDAC) enzymatic activity, which mediates the removal of acetyl groups and therefore goes along with chromatin compaction and transcriptional inactivation. HDACs are grouped into four classes according to their relationship to their homologues in *S. cerevisiae*: class I (HDAC1-3, HDAC8), class II (HDAC4-7, HDAC 9–10), class III sirtuins (SIRT1-7), and class IV (HDAC11) [22]. With the exception of class III HDACs – sirtuins, which are NAD^+^-dependent, all other HDACs are Zn^2+^ dependent. Both, HATs and HDACs unspecifically influence the acetylation status of proteins. Their specificity as to the posttranslational modification of histone proteins may – at least in part - be achieved through their recruitment to chromatin within multi-protein complexes, [22] and their localization within specific cellular compartments. Massive acetylation of lysine residues may activate transcription through neutralization of the basic charge of these residues and through the recruitment of bromodomain-containing protein complexes, which may include other HATs and chromatin remodeling enzymes [25]. This process goes along with an easily accessible chromatin configuration, so that transcription is facilitated [26]. The acetyl groups that are needed for this process are transferred from acetyl-coenzyme A complexes, and the reaction is catalyzed by three principal families of HATs: CBP/p300, GNAT and MYST [1]. The different families of mammalian HDACs are grouped in four classes: class I (HDAC1-3, HDAC8), class II (HDAC4-7, HDAC 9–10), class III sirtuins (SIRT1-7), and class IV (HDAC11) [22,27,28].

### Histone methylation

Unlike the acetylation of histone proteins, the methylation of histone lysine and/or arginine residues has variable effects on gene expression. The methylation of histone lysine residues has been extensively studied and in fact, the effect on gene expression very much depends on the specific lysine residue that is being modified. Also, the single lysine residues can be variably methylated to mono-, di- and trimethylated states [2,29,30]. Every such status is essential and allows a highly specific distinction concerning the methylation pattern: active promoters for example, are enriched in trimethylated H3 lysine 4 (H3K4) residues, whereas enhancer elements are enriched in monomethylated H3K4 residues [31]. On the other hand, di- and trimethylated histone H3 lysine 9 (H3K9) residues are strongly correlated with transcriptional repression [22,32]. Enzymes that catalyze this reaction, act dynamically as either histone methyltransferases or histone demethylases. e.g. lysine-specific histone demethylase 1 which demethylases H3K4 or Jumonji C (JmjC)-domain-containing family of proteins which catalyse demethylation of H3K4me3 [29]. Few lysine residues can be either methylated or modified by acetylation, but never both together [2].

### RNA-based mechanisms

So far, the RNA-based mechanisms are the least well understood mechanisms of epigenetic regulation. Noncoding RNA (ncRNA) appear to be involved in the chromatin-based regulation of gene expression [2,6,33]. In fact there is increasing evidence that RNA-based mechanisms combine the coordinated activities of this noncoding RNA together with other epigenetic modifications, such as DNA methylation and posttranslational histone modifications. For example the RNA-induced initiation of transcriptional gene silencing complex (SISC) forms heterochromatic sites as a consequence of histone methylation[34]. Similarly, the expression of ncRNA itself may be modulated through epigenetic mechanisms: miRNA 124 for instance, is silenced in different types of cancer by DNA-methylation. These are only two examples for the complexity and intertwinement of epigenetic mechanisms. NcRNAs are defined by their number of nucleotides: Short ncRNAs count up to 200 nucleotides long ncRNAs count more than 200 nucleotides [2]. These ncRNAs seem to regulate the chromatin compaction state of defined genomic loci [35]. Large intervening non-coding RNAs (lincRNAs) are able to recruit chromatin modifying complexes and/or to overlap the coding region of genes and thus to regulate gene expression at the level of specific target loci [2,36]. As an example, Khalil et al. found about 20% of 3,300 human long intergenic ncRNAs were bound by Polycomb Repressive Complex 2 (PRC2) or other chromatin-modifying complexes [36]. In addition, long ncRNAs (>200 nucleotides) are able to mediate transcriptional activation by recruiting the H3K4 mixed-lineage leukemia methyltransferase (MLL1) [35]. Small non-coding RNAs on the other hand, are ~21 bp long with target messengers that can induce degradation of the dsRNA complex through the action of the RNA III endonuclease (also referred to as “Dicer”) [37]. The transcriptional silencing by small ncRNAs like microRNAs (miRNA) and short interfering RNAs (siRNA) is currently being investigated very intensively due to their promising therapeutic potential. These small RNAs are grouped into different classes depending on their length and function [38]. They are able to specifically inhibit target mRNAs of different genes. MiRNAs and siRNAs are 21 to 26 nucleotides long, and are well-known mediators of tissue-specific posttranscriptional gene silencing as a part of RNA-induced silencing complex (RISC) and as siSC (scrambled inhibitory RNA) respectively [1,37] miRNAs are synthesized by RNA polymerase II as a long RNA primary transcript (*pri-miRNA)* and cleaved by Drosha, the double-stranded RNA endonuclease III, to produce a typical stem-loop structure, known as a *pre-miRNA*[39]. The nuclear RNase III Drosha initiates microRNA processing [39]. Drosha is part of a multi-protein complex, which contains the double-stranded RNA binding protein Pasha also known as DGCR8 [40]. Pasha binds single-stranded pri-miRNA fragments and stabilizes them for processing by Drosha. Additional enzymes that promote the maturation of miRNA are two proteins called Argonaute and Dicer which are part of RISC. Drosha and Pasha are both localized within the cell nucleus, where the processing of pri-miRNA to pre-miRNA takes place. This pre-miRNA is then exported into the cytoplasm via Exportin 5 and then further processed by the RNase Dicer to dsRNA which is then transported back into the nucleus via RISC. The mature single strand (‘guide strand’) is paired to the 3’-untranslated region of the target RNA and leads to its degradation [41]. SiRNAs are derived from long double-stranded RNA precursors and may be found in cells naturally or the may be exogenously imported and in fact, transcriptional gene silencing can be achieved by exogenously administered siRNAs directed to promoter regions in mammalian cells [1]. The mechanisms that are involved herewith are site-specific DNA methylation [42] and repressive histone posttranslational modifications [43]. In the future, studies might demonstrate the far-reaching effects of RNA-based mechanisms on the regulation of mammalian gene expression.

## Epigenetics of blood vessel physiology

During development and lifelong sustainment of the vascular system epigenetic factors play a crucial role and permit a high flexibility to sudden physiological changes.

### Epigenetic mechanisms in vascularization

After fertilization, the vascular system is one of the first organ structures that develop. During “vasculogenesis” a primary capillary plexus is formed *de novo* from mesodermal cell precursors, while the formation of new vessels from pre-existing vessels is referred to as “angiogenesis” and is based on the interaction of pro- and anti-angiogenic molecules [44]. The initiation of vascular development depends on the presence of Fibroblast growth factors in order to induce hemangioblastic differention. In a next step vascular endothelial growth factor (VEGF) further triggers cell differentiation. The formation of a capillary plexus strongly depends on the expression of adhesion molecules which form inter-cellular connections such as VE (vascular endothelial) cadherin, N-cadherin and connexins, as well as molecules which promote cell matrix interactions (netrins, semaphorins, fibronectin, integrins) and a number of signaling pathways that include key proteins such as NOTCH, VEGF1/2, transforming growth factor beta (TGF-ß), or ephrin type-A receptor 2 (Eph-2) and 4, only to mention a few [45,46]. The acetylation of Notch via SIRT1 regulates the amplitude and duration of Notch activity and allows for normal vascular sprouting [47]. In a second stage, haemodynamic factors and local hypoxia model the structure, identity and function of the blood vessels, [48-50] which accentuates the role of epigenetic mechanisms [4]. In summary, epigenetic mechanisms strongly influence the development of the vascular system [1,51].

Vascular development, as well as endothelial and smooth muscle cell differentiation and function, require a fine epigenetic adjustment. The promoter regions of several cell-specific endothelial proteins such as the endothelial nitric oxide synthase (endothelial isoform of NO synthase, eNOS [alias: NOS3]) are constitutively active [52] and it appears that the key difference to other cell populations that do not express eNOS is observed at the chromatin level: while a relaxed and thus transcriptionally permissive chromatin structure may be found at the level of the eNOS promoter in endothelial cells, this very locus appears to be highly condensed and thus in a repressive configuration in non-endothelial cells [2]. In addition, the expression of the human eNOS-gene is at least in part, also determined by the DNA methylation status [53] Matouk et al. proposed that additional chromatin-based mechanisms could be relevant for the cell-specific expression of eNOS. In non-expressing cell types, the eNOS promoter was hypermethylated and lacked activating histone posttranslational modifications, whereas this very promoter was reported to be hypomethylated and enriched with activating posttranslational histone modifications such as acetylated H3K9, H4K12 as well as di- and trimethylated H3K4 in endothelial cells [53,54]. Endothelial-specific transcription factors such as KLF2 are also involved in the regulation of the tissue specific expression of endothelial proteins, [55] moreover, KLF2 is not even responsible for the differentiation of precursor cells to endothelium which underlines the significance of epigenetic regulation [4].

Several studies show that HDAC activity mediates angiogenesis as a consequence of hypoxia, [56] shear stress- and VEGF-induced stem cell differentiation [57,58]. On the other hand the inhibition of HDAC prevents the proliferative response to VEGF [59]. There is some evidence that the global and endothelial cell-specific knockout of HDAC 7, a class II HDAC, is associated with embryonic lethality and required for the development of a normal vasculature [60]. HDAC7 is an important effector molecule in the context of the VEGF-signaling pathway: the suppression of HDAC7 phosphorylation and subsequent translocation to the cytoplasm inhibits VEGF-mediated proliferation and migration [61]. HDAC activity is known to be critical for the endothelial differentiation of embryonic stem cells as well as adult endothelial progenitor cells [57,58,62,63]. Histone acetylation controls the expression of important signaling molecules like the von-Willebrandt-factor, NOTCH and eNOS [52]. Nevertheless, for the sprouting process during angiogenesis, histone methylation appears to be very important [64].

Several studies correlated environmental stimuli during development with non-Mendelian diseases, such as cardiovascular disease. The persistence and reproducibility of these studies indicate permanent non-genomic modifications, which result in a certain cellular “re-programming”. The modulations needed for this might be based and – at least in part - explained by epigenetic mechanisms [4]. Such studies show that epigenetic modulations also reflect environmental signals under physiological and pathological conditions [65,66]. However, only little information is available on the varying response in foetal endothelial cells in the context of different pregnancy related diseases [67,68]. Important evidence for the immediate role of epigenetic mechanisms in pathological alterations is found in diabetes. In this context, it has already been shown how intra uterine growth restriction (IUGR) generates abnormal programming of gene expression at different levels, which could advance the development of insulin resistance and/or type 2 diabetes [4].

### Postnatal angiogenesis

During the postnatal period, HDACs have been reported to be key regulators of angiogenesis [1]. Accordingly, HDAC inhibitors have already proven to exhibit potent antiangiogenic activity [69]. Some HDACs appear to have prominent roles in this context: HDAC1 for example plays a pivotal role in hypoxia-induced angiogenesis [70]. On the other hand, specific knockdown of HDAC7 constrains cell migration and angiogenesis in mature primary endothelial cells in culture [71]. Another important factor in angiogenic signaling is constituted by SIRT1, a class III HDAC [72]. SIRT1 is highly expressed during blood vessel growth and allows for sprouting during angiogenesis through deacetylation of the forkhead transcription factor Foxo1 [73].

So far, little information is available on what is going on within the specific angiogenesis pathways, but it is likely that the acetylation status of various transcription factors that are being involved plays an important role [1].

### Shear stress

Laminar flow may influence gene regulation via epigenetic pathways and thus, disturbed flow is capable of changing gene expression in cells [2]. Shear stress for instance, enhances histone H3 K79 methylation in mouse embryonic stem cells (ESC), [58] where shear stress causes global histone modification changes and promotes ESC differentiation to an endothelial cell lineage [58,74]. Accordingly, laminar shear stress also goes along with both, global and gene-specific histone modifications in cultured human endothelial cells [75]. Shear stress enhances the activity of HAT p300 in human endothelial cells and leads to the acetylation of H3 and H4 at the level of the eNOS promoter among others [30] HDACs can also modify gene expression in response to shear stress for example through the VEGF-signaling pathway: Zeng et al. showed that HDAC3-mediated p53 deacetylation and p21 activation caused by shear stress and VEGF induces differentiation of endothelial cells through the VEGF receptor 2 (Flk-1)–PI3K–Akt signal pathway [57].

### Hypoxia

Intrauterine hypoxia i.e. lower oxygen tension in the fetus compared to the adult, is to a certain degree essential for cardiac formation. Hypoxia decreases global gene expression via epigenetic pathways: A global decrease in H3K9 acetylation can be observed in various types of cells as a consequence of increased HDAC activity [76,77]. On the other hand, acetylated H3K9 is found at the promoter level for hypoxia activated genes such as VEGF [76-78]. Increasing evidence also accumulates that the hypoxia- inducible factor (HIF-1α) is involved in the maintenance of global transcriptional silencing, as well as in directing gene repression to specific genes [79,80] Kato et al. showed that HDAC7 is transported to the nucleus together with HIF-1α under hypoxic conditions where it upregulates the transcriptional activity of HIF-1α forming a complex together with p300 [80]. In conclusion, the current understanding of hypoxia-induced epigenetic changes is comparatively poor and needs further investigation.

## Cardiovascular disease

In the past few years, there has been increasing evidence that part of the gene-environmental interactions is regulated by epigenetic mechanisms. Accordingly, abnormal regulation in this context can result in complex diseases.

### Atherosclerosis and restenosis

**Figure 1:**
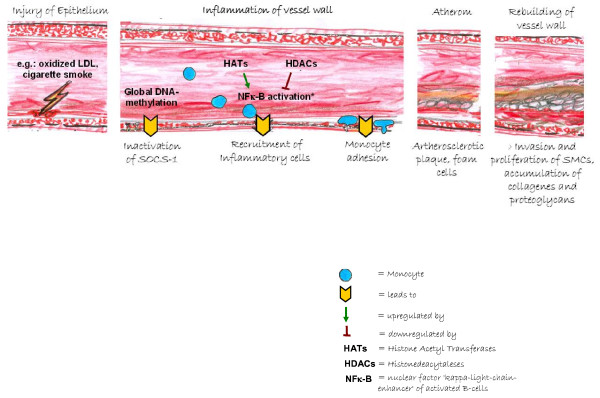
**Pathogenesis of Atherosclerosis.** Injury of vascular epithelium by oxidized low density lipoproteins (oxidized LDL) or cigarette smoke leads to inflammation of tissue and global DNA-methylation within the cells. Proinflammatory transcription factors like NFκ-B are activated subsequently and expression of endothelial adhesion molecules leads to monocyte adhesion. These cells incooperate large quantities of oxidize d LDLs and become foam cells (forming of atherom). The forming of an atherosclerotic plaque is accompanied by the invasion of the vessel wall by smooth muscle cells (SMCs) and the accumulation of collagens and proteoglycans (Rebuilding of vessel wall).

**Figure 2:**
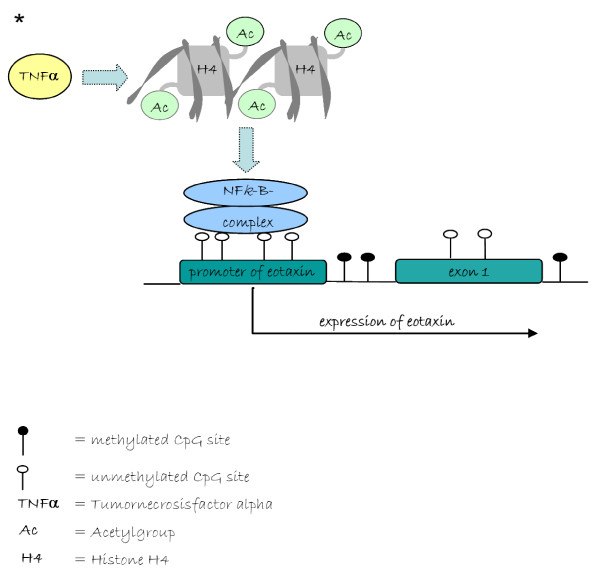
**Epigenetic modulation of Eotaxin expression via NFκ-B.** Inflammatory signalling via TNF-α leads to acetylation of histone H4 and induces the binding of p65 subunit of NFκB-complex to unmethylated sites of the eotaxin promoter. Transcription of the eotaxin gene is induced and consequently the number of inflammatory cells which are recruited and activated within the atherosclerotic plaque.

Some of the genes that are regulated by epigenetic modification constitute a major part of the regulatory work of extracellular matrix formation, inflammation and proliferation, which are involved in cardiovascular pathology including atherosclerosis and restenosis [3]. Both, atherosclerosis and restenosis are intensively affected by the inflammatory response to endothelial injury and the subsequent reshaping of the vessel wall in size and composition, also referred to as “remodelling” (Figure 1) [81,82]. For both disorders the proliferation and migration of vascular smooth muscle cells (VSMCs) and the formation of extracellular matrix, leads to an accumulation of collagen and proteoglycans, which finally results in to the occlusion of blood vessels [3]. For atherosclerosis, elevated lipoprotein levels and cigarette smoke are a main effectors, [83] while restenosis is mainly an overshooting wound healing process in response to vascular injury by balloon dilation or stent placement. Atherosclerosis develops from the accumulation of oxidized lipoproteins within foam cells and extracellularly together with the proliferation of arterial smooth muscle cells. The incidence of atherosclerosis is associated with increasing age, whereas restenosis is develops rapidly after interventions of revascularization [3] Global DNA hypermethylation appears to be significantly associated with vascular inflammatory response to endothelial injury and correlates with cardiovascular mortality [84] The underlying mechanisms that lead to inflammation are believed to be based on the inactivation of suppressors of cytokine signaling (SOCS) [85]. Atherosclerosis and restenosis are worsened as a consequence of multiple inflammatory pathways, which are regulated by pro-inflammatory transcription factors like NFκ-B [3,86]. The activity of NFκB is known to be regulated through posttranslational acetylation, i.e. by HATs and HDACs [87]. Eotaxin, which is a chemoattractant for eosinophilic granulocytes, is an important player in the context of inflammatory response and appears to be a target of epigenetic regulation: inflammatory signaling via TNF-α specifically leads to histone H4 acetylation and induces the binding of p65 to the eotaxin promoter, which induces transcription of the eotaxin gene and consequently, increasing numbers of inflammatory cells are being recruited and activated within the atheromatous plaque (Figure 2) [88,89]. Another factor that plays an important role in atherosclerosis and restenosis is the granulocyte macrophage colony-stimulating factor (GM-CSF). In a rabbit model, GM-CSF injections have been shown to reduce the formation of neointima and in accordance with a patient study, NFκB-mediated inflammatory signaling concurs with histone H4 hyperacetylation, which correlates with increased GM-CSF expression levels [3,90]. The predominant finding in this study was a reduction in the progression of artherosclerosis by GM-CSF.

Furthermore the HDAC inhibitor trichostatin A (TSA) increases the expression of GM-CSF in alveolar macrophages and airway epithelial cell lines after activation with inflammatory stimuli, [91-93] which suggests that the signalling pathway that involves GM-CSF could affected by alterations at the epigenomic level. Numerous other factors such as cyclooxygenase-2 do also appear to be regulated via epigenetic modifications. Taken together, epigenetic modulation seem to play a key role in the regulation of inflammatory events and could therefore become important structures of targeted medical therapy.

### Myocardial infarction

In a murine model of ischemia and reperfusion, ischemia was shown to induce HDAC activity in the heart and consequently deacetylation of histones H3 and H4 [94]. From 1 h prior to ischemia until 45 min after reperfusion, the HDAC inhibitors TSA and scriptaid were able to reverse the activity of ischemia-induced HDACs in vivo and to reduce myocardial infarct size by up to 50%. In an in vitro study, it has been shown that a 5 h period of hypoxia resulted in a strong decrease in acetylated H3 and H4 histones in mouse cardiomyocytes, which was completely blocked by TSA [94]. In 2002 Mc Kinsey et al. found that class IIa HDACs (i.e. HDAC4, -5, -7, and −9) interact with members of the myocyte enhancer factor-2 (MEF2) transcription factor family which are key regulators of cardiac hypertrophy. Therefore, the down regulation of these HDACs raises new possibilities as to cardiovascular disease treatment and prevention [95] Zhang et al. demonstrated that the inhibition of histone deacetylases (HDAC) protects the heart against ischemia-reperfusion (I/R) injury in which the acetylation of NFκBp50 plays the pivotal role [96]. In summary, epigenetic pathways play an important role in myocardial infarction, and HDAC inhibitors could therefore open new horizons as to improved individualized treatment strategies in the future.

### Abdominal aortic aneurysm

Abdominal aortic aneurysm (AAA) appears to be the result of an imbalance between aortic extracellular matrix destructive and restorative processes [8]. In this context, the normal lamellar architecture of the aorta gets destroyed and is subsequently invaded by inflammatory cells, including T/B lymphocytes, macrophages, neutrophils, mast cells and plasma cells [97]. In animal models, AAA have been shown to be a dynamic remodelling process, with neovascularisation, inflammatory cell infiltration, endothelial dysfunction, apoptosis and depletion of the vascular smooth muscle cells, as well as destruction of the elastic media [98]. The pathogenesis of AAA is poorly understood, and there seem to be multiple environmental and genetic factors which cause the initiation and progression of AAA. Many of the AAA risk factors including cigarette smoke, older age, male gender and hypertension, have already been linked to epigenetic effects and could promote AAA. Previous investigations demonstrated that persistent inflammation in the context of atherosclerosis might lead to aberrant DNA methylation as well as alterations in histone modification. Given that AAA has a strong inflammatory component, it is likely that epigenetic mechanisms are involved in the progression of AAA [8]. In human AAA biopsies, the matrix metalloproteinase-2 and 9 (MMP) whose proteolytic activity contributes to matrix degradation in AAA, are significantly upregulated and the expression of these MMPs is in fact regulated by posttranslational acetylation at the histone level [8,99,100].

### Raynaud’s syndrome

Even more than 100 years after its first description, the pathogenesis of Raynaud’s syndrome is still quite unclear [101,102]. The interaction of different regulatory mechanisms between endothelium, smooth muscle and autonomic and sensory innervation, which ultimately lead to the pathological vasospasm of this disease, is extremely complex and affects both, endothelium-dependent and endothelium-independent mechanisms of vasoregulation. The endothelial vasoregulation is based on the interplay between opposing vasoconstrictive mediators (e.g. plasma endothelin-1 [EDN1], thromboxane [TXA 2], methyl arginine, transcription factor HIF-1α [hypoxia-inducible factor 1-alpha] and acetylcholine) and vasodilating agents (e.g. prostaglandin [prostacyclin], CGRP [calcitonin gene-related peptide] and eNOS [102-104]. The activity of eNOS - as an example for a gene that is being regulated at the epigenomic level - can be reduced by histone deacetylase (HDAC) inhibitors, which consequently results in reduced vasorelaxation [52]. Furthermore, there are direct interactions between epigenetic regulators and proteins, being involved in the regulation of the vascular tone, e.g. the transcription factor HIF-1α, which interacts directly with histone deacetylases [80].

Importance of NO, NADPH/NADH oxidase in the modulation of vascular tone

The vascular endothelium is not only a structural semi-selective diffusion barrier between the vessel lumen and the interstitial space and contributes to the modulation of inflammation and coagulation, but also regulates blood pressure, vascular tone and blood flow by the release of a large number of vasodilating substances such as nitric oxide (NO) and prostacyclin (PGI2) and of vasoconstrictors such as endothelin (ET) and platelet factor (platelet-activating factor (PAF)) [105-107]. In addition to its role as a vasodilator, there are a number of other biologically important effects of NO: NO improves the perfusion of the coronary arteries and the cardiovascular system. It can lead to swelling of the corpus cavernosum of the penis and can relax the vascular muscle and the bronchial tree. NO is released from the endothelial cells and inhibits prostacyclin in combination with the aggregation of platelets [108]. Nitric oxide (NO) is present in almost every organ system and is produced by the endothelial NO synthase by the cleavage of the amino acid L-arginine. Because of its short half-life, it is generated directly in the target area. eNOS is expressed mainly by the vascular endothelial cells of the tunica media of the arterial blood vessels. Its production is influenced by various physiological and pathophysiological stimuli [109,110]. The expression of eNOS is regulated at the transcriptional and at the posttranscriptional level. Meanwhile, several physiological and pathophysiological stimuli, influencing the transcription of the eNOS gene, are known: Thus, the mRNA expression of eNOS is significantly increased in response to lysophosphatidylcholine, shear stress and TGF-beta [111]. The eNOS mRNA level not only depends on the transcriptional activity at the promoter level, [111]. the post-transcriptional modulation of the eNOS mRNA half-life is probably the determining factor that influences the actual amount of eNOS mRNA. eNOS mRNA can be stabilized through exposure of endothelial cells to 3-hydroxy-3-methylglutaryl (HMG)-CoA reductase inhibitors, [112] the VEGF or the exposure to shear stress [113,114].

TNF-alpha, [115] oxLDL, [116] hypoxia, [116] and cell confluence, [116] however, accelerate the degradation of eNOS mRNA. The expression of eNOS mRNA is pathological in a variety of vascular disorders [111]. A decrease of the expression of eNOS mRNA has been described in cardiovascular diseases [54].

The half-life and biological activity of NO is decisively affected by reactive oxygen derivatives (ROS), such as O_2_[117,118] O_2_ and nitric oxide (NO) are important substrates as to the regulation of vasodilation and vasoconstriction. These small molecules exhibit opposing effects on the vascular tone and result in the production of potentially toxic substances, such as peroxynitrite (ONOO-) [119-125]. The endothelial bioavailability of NO in atherosclerosis and in heart failure is reduced through a decreased activity of antioxidant enzyme systems (ecSOD = extracellular superoxide dismutase) and increased activity of oxygen radical-producing enzyme systems (NADPH oxidase, xanthine oxidase). In endothelial cells and in vascular smooth muscle cells, this membrane-associated, NAD(P)H-dependent oxidase is the most important O_2_-producing enzyme in endothelial cells, smooth muscle cells and/or vascular adventitia. There may be a link between the NAD(P)H- dependent oxidase NAD, being required by sirtuins. It is reported that the eNOS activity can be reduced by HDAC inhibitors, resulting in an impaired relaxation of blood vessels [52].

In summary, the endothelium-dependent vasoregulation depends on the interplay between opposing vasoconstrictive (e.g. plasma endothelin-1 (EDN1), thromboxane (TXA2), methyl arginine, transcription factor HIF-1α and acetylcholine), and vasodilating activities (e.g. prostaglandins (prostacyclin), CGRP (calcitonin gene related peptide) and the endothelial isoform of nitric oxide synthase (eNOS, synonym: NOS3)) [102-104].

### The role of non-coding RNAs in cardiovascular disease

MicroRNAs (miRNAs) belong to the group of non-coding RNAs and work as regulators of gene expression at the mRNA level by suppression of translation and finally degradation of mRNAs for example by Ago2 mediated cleavage [38]. The expression profile of miRNAs in a rat model of vascular restenosis showed that 100 out of 140 miRNAs were differentially regulated during neointima reformation. In this context miRNA 21 was identified to be a key regulator of cell proliferation and the redifferentiation of smooth muscle cells (SMCs) [126]. Downregulation of miRNA 145 and miRNA 143 after vascular injury modulates the SMC cytoskeleton at least in part by induction of KLF5, which further reduces SMC contractility. SMC proliferation is – among other things - enhanced by the downregulation of cell cycle inhibitors such as p27 and p57 and by increased endogenous levels of miRNA 221 and miRNA 222 [127]. The inflammation of vessel walls during the development of atherosclerosis is enforced by the downregulation of miRNA 126 which promotes the expression of VCAM-1 on one hand, while it induces the production of CXCL12 on the other leading to the recruitment and adhesion of inflammatory cells [128,129]. As reported by Menghini et al. an increased expression of miRNA 217 in atherosclerotic plaques in patients leads to the disintegration of the endothelium and thus to the acceleration of vascular senescence via inhibition of SIRT1 [130]. Since miRNAs appear to be of utmost importance in the pathogenesis of cardiovascular disease, the profiling of circulating miRNAs might be a useful biomarker for disease assessment [131]. Fichtlscherer et al. confirmed the reduction of circulating miRNA 126 and miRNA 145 amongst others in the serum of patients with coronary artery disease [132]. Other miRNAs that qualify as biomarkers are miRNA 1, miRNA 133b and miRNA 499 which have been shown to be elevated in patients and animal models of acute myocardial infarction [133,134].

LncRNAs, non-coding RNAs with more than 200 nucleotides, are transcribed as overlapping sense and anti-sense transcripts to coding DNA regions regulating the transcription of corresponding overlapping mRNA [38]. Robb and colleagues found evidence for the regulation of eNOS expression by overlapping antisense lncRNA, [135] which was upregulated during hypoxia [136]. Also, hypoxia induces the antisense transcript to the 3′ UTR of HIF −1α which is similarly expressed in renal cancer and different human tissues [137,138].

Non-conding RNAs play a pivotal role in the pathogenesis of cardiovascular disease and offer the possibility to operate as diagnostic and prognostic biomarkers.

## Perspectives for treatment and prevention

Unaltered epigenetic regulation pathways like DNA methylation, posttranslational chromatin modifications and ncRNAs provide the basis for healthy cardiovascular system. By now, many studies show positive effects and possibilities of treatment via epigenetic modulators. Based on these experiences therapeutic intervention of hypoxia associated diseases like asthma and chronic obstructive pulmonary disease as well as of inflammatory processes by modulation of epigenetic factors seems to be promising.

Nutritional factors are highly essential in the prevention of cardiovascular disease and comprise both, the avoidance of unfavourable food supplements such as high concentrations of low density lipoproteins and salt as well as the accentuation of beneficial effects of nutritional ingredients such as folic acid or resveratrol [139-143].

In 1993 Frankel et al. identified antioxidative effects for resveratrol (3,4′,5-trihydroxystilbene) on low density lipoproteins (LDLs) thus reducing a crucial initiation event in the pathogenesis of atherosclerosis [144]. In addition, Wallerath et al. found evidence of resveratrol enhancing the activity and expression of eNOS in vitro [145]. In 2006, resveratrol, which is a plant derived activator of SIRT1, was found to decrease reactive oxygen species, inflammation, and apoptosis in the aortas of elderly mice that ran through a high-fat diet. Resveratrol is known to maintain normal endothelial function [146]. In transgenic apolipoprotein E null mice overexpression of SIRT1 was reported to reduce atherosclerosis due to increased endothelial cell survival and function [147]. In addition, SIRT1 was demonstrated to prevent excessive superoxide production and to reduce inflammation through inhibition of NFκB signalling and a reduction of ICAM-1 and VCAM-1 expression levels [148]. SIRT1 also induces and directly enhances the expression of eNOS, which decreases the speed of vascular senesce. The activation of SIRT1 therefore appears to be an important tool as to the therapy of cardiovascular disease.[149,150] The regulation of SIRT3 is closely connected to SIRT1 and seems to protect against damage caused by myocardial infarction or chronic heart failure [151]. Resveratrol is a plant derived factor that is mainly found in berries, grape skin and red wine and was the first activator of SIRT1 to be identified [145]. Very recently it was shown in a mouse model that resveratrol prevents diet induced left ventricular hypertrophy as well as interstitial fibrosis and diastolic dysfunction {Qin, 2012 #16820}. Resveratrol represents the tip of the iceberg when it comes to the effects of nutrition on the epigenome and thus, their role in the prevention and pathogenesis of cardiovascular disease.

Folates are part of the family of water-soluble B vitamins, which directly serve the one-carbon-metabolism with methionine for the production of S-adenosylmethionine. This in turn is the one-carbon-donor for the methylation of DNA [152]. Folic acid deficiency leads to global DNA hypomethylation which comes along with an increased risk for cancer and cardiovascular disease [70,139]. During the course of atherosclerosis smooth muscle cells transform and share a number of characteristic similarities with cancer cells such as the overexpression of proto-oncogenes, growth-factors as well as increased cell motility [142]. This transformation is based on the global hypomethylation of DNA. Accordingly, at the age of four weeks ApoE mice show changes in the DNA methylation pattern prior to the generation of atherosclerotic lesions [153]. Similarly, a reduction of ~ 9% in C5-methylation was identified in advanced atherosclerotic plaques in humans [154]. Folic acid directly influences the function of vascular endothelial cells via improving NO production and vasodilation. Doshi et al. for example showed improvement of endothelial function in CVD patients after intervention with homocysteine {Doshi, 2002 #16819}.

Resveratrol and B vitamins prove to be important both, in the prevention and the treatment of cardiovascular disease and carry the advantage of being practically free of side effects. Currently more than hundred clinical trials are ongoing or already completed studying the aspects of resveratrol or folate administration in subjects thus emphasizing their relevance for prevention of diet induced CVDs in humans (ClinicalTrials.gov).

In addition to nutritional factors a number of small molecule HDAC inhibitors for the treatment of cardiovascular disease are currently available:

In preclinical studies, most of the small molecule inhibitors of HDACS are hydroxamid acid derivates like the pan-HDAC inhibitor TSA and suberoylanilide hydroxamic acid (SAHA) [8].

HDAC inhibitors (HDACIs) are potential candidates in the treatment of cardiovascular disease such as cardiac hypertrophy, heart failure, ischemia/reperfusion injury, atherosclerosis and restenosis [155,156], Gallo et al. for example demonstrated in a mice model that the selective inhibition of class I HDACs with an apicidin derivate prevented cardiac hypertrophy and failure [157]. Rajasingh et al. treated mouse bone marrow progenitor cells with TSA and 5-Aza and further differentiated these cells into myocyte progenitors. The implantation of these cells led to an improvement of cardiac function after infarction. This points out another route small molecules unclose for CVD treatment {Rajasingh, 2011 #16828}. In recent in vitro experiments TSA was shown to agonist load- and agonist-induced hypertrophy which suppressed autophagy of myocytes. These data where supported by the finding that the ventricular mass of hypertrophic animals was normalized as well as ventricular function {Cao, 2011 #16832}.

In the treatment of AAA, there have recently been new results: Metacept-1 (MCT-1), a synthetic derivate of oxamflavin, also serves as an inhibitor of HDAC and showed AAA inhibitory effects in a mouse model [158]. HDAC inhibitors seem to constitute a potential way of managing AAA by targeting aortic proteolytic degradation.

## Conclusions

In summary, class II and class III HDACs where shown to exert heart protective activities [95]. Further investigations are crucial to get a better understanding of the complex epigenetic interactions and to provide new ways for the treatment of vascular disease.

## Abbreviations

AAA, Abdominal aortic aneurysm; CGRP, Calcitonin gene related peptide; DNMTs, DNA methyltransferases; ecSOD, Extracellular superoxide dismutase; EDN1, Plasma endothelin-1; eNOS, Synonym: NOS3, endothelial isoform of nitric oxide synthase; ESC, Embryonic stem cells; ET, Endothelin; GM-CSF, Granulocyte macrophage colony-stimulating factor; HATs, Histone acetyltransferases; hCGBP, Human CpG binding protein; HDAC, Histone deacetylase; HDACIs, HDAC inhibitors; HIF-1, Hypoxia-inducible factor; HIF-1, Hypoxia-inducible factor 1-alpha; IUGR, Intra uterine growth restriction; lincRNAs, Large intervening non-coding RNAs; MEF2, myocyte enhancer factor-2; miRNA, microRNAs; MMP, Matrix metalloproteinase; ncRNA, Noncoding RNA; NO, Nitric oxide; ONOO-, Peroxynitrite; PAF, Platelet-activating factor; PRC2, Polycomb repressive complex 2; RISC, RNA-induced silencing complex; ROS, Reactive oxygen derivatives; SAHA, Suberoylanilide hydroxamic acid; siRNA, Short interfering RNAs; SMCs, Smooth muscle cells; SOCS, Suppressors of cytokine signalling; TGF-ß, Transforming growth factor beta; TSA, Trichostatin A; TXA2, Thromboxane; VEGF, Vascular endothelial growth factor; VSMCs, Vascular smooth muscle cells.

## Competing interests

The authors declare that they have no competing interests.

## Authors’ contributions

All authors have written and edited the manuscript. All authors read and approved the final manuscript.
